# A Novel 13 bp Deletion within the *NR6A1* Gene Is Significantly Associated with Growth Traits in Donkeys

**DOI:** 10.3390/ani9090681

**Published:** 2019-09-14

**Authors:** Xiya Fang, Zhenyu Lai, Jie Liu, Chunlan Zhang, Shipeng Li, Fei Wu, Zihui Zhou, Chuzhao Lei, Ruihua Dang

**Affiliations:** Key Laboratory of Animal Genetics, Breeding and Reproduction of Shaanxi Province, College of Animal Science and Technology, Northwest A&F University, Yangling, Xianyang 712100, China; fxiya5819@163.com (X.F.); lzy18408210600@126.com (Z.L.); SCLJ0562@163.com (J.L.); zcl253873373@163.com (C.Z.); lsp17782767206@163.com (S.L.); 18392360732@163.com (F.W.); 15636110299@163.com (Z.Z.); leichuzhao1118@126.com (C.L.)

**Keywords:** body height, body length, donkey, *NR6A1*, polymorphism

## Abstract

**Simple Summary:**

The detection of genes potentially associated with economic traits and identification of effective variants can provide a basis for molecular marker-assisted selection of livestock. NR6A1 is a member of the nuclear receptor family and is an important candidate gene related to body size traits. Previous studies showed that *NR6A1* gene was associated with body size traits in pigs and other livestock, however, it has not yet been observed in donkeys. In the current study, a 13 bp deletion in *NR6A1* gene was firstly identified in donkeys. Analysis showed that this deletion had significant associations with body size traits.

**Abstract:**

Nuclear receptor subfamily 6, group A, member 1 (NR6A1), as an important member of the nuclear receptor family, plays an important role in regulating growth, metabolism, and differentiation of embryonic stem cells. For this reason, the *NR6A1* gene is considered to be a promising candidate for economic traits and was found to be associated with body size traits in many livestock. However, no studies have been conducted on *NR6A1* in donkeys so far. Thus, in this research, we focused on donkeys and identified a 13 bp deletion in intron-1 of the *NR6A1* gene among 408 individuals from Guanzhong and Dezhou donkeys using polyacrylamide gel electrophoresis. Three genotypes were identified, namely II, ID, and DD. The association analysis indicated that the body lengths and body heights5f genotype II individuals were significantly different to those of genotype ID in Dezhou donkeys. Conclusively, the 13 bp deletion was associated with growth traits in both Guanzhong donkeys and Dezhou donkeys, indicating that the *NR6A1* gene could be a possible candidate gene in marker-assisted selection for donkey breeding programs.

## 1. Introduction

Body size is one of the most important economic traits of livestock. Length and number of vertebrae are also influential determinants of body size traits. More ribs mean longer bodies [1,2,3], and the variation in vertebral number is mainly influenced by genetics, so the vertebral trait has high heritability [4]. Some polymorphic loci identified in the *ZBTB38* gene and the *NPY* gene in Chinese cattle (Nanyang, Qinchuan, et al.) and the *UCP3* gene in Simmental Hybrid Cattle [5] were confirmed to be associated with body length, body height, rump length, and other body size traits [6,7], which can be used as molecular markers in cattle breeding. As a critical member of the nuclear receptor family, NR6A1 is highly expressed in the developing nervous system, placenta, and developing germ cells, and plays an important role in the early development of the embryo [8,9]. The *NR6A1* gene of donkeys is located from 2,209,314 to 2,264,416 (NW_014637488.1, Unplaced Scaffold) and is 55,102 bp in length with 11 exons. Several studies showed that *NR6A1* was indeed associated with the number of vertebrae in pigs and other species [3,10]. Genome-wide association studies revealed that the *NR6A1* gene was related to the number of vertebrae in pigs [3]. Polymorphisms in *NR6A1* in sheep also influence the number of lumbar vertebrae [10].

Some researchers [11] found that the inhibitory effect of NR6A1 on embryonic multi-potential genes was necessary for the process of embryonic stem cell differentiation, which is induced by retinoic acid. They put forward that embryonic stem cell self-renewal and directional differentiation was closely related to the expression of *NR6A1*. *POU5F1* is a key gene for the maintenance of pluripotency of embryonic stem cells. NR6A1 is the first known factor to inhibit *POU5F1* and was found to promote *POU5F1* gene methylation, resulting in *POU5F1* gene silencing thereby inhibiting its expression [12] and depriving stem cells of pluripotency. A series of research work revealed the association of quantitative trait loci (QTLs) on chromosomes 1 and 7 with the increased number of vertebrae in European wild pigs compared to Asian wild pigs [13]. In these, a causative mutation in *NR6A1* located on chromosome 1 was found to be one of the reasons behind variable vertebrae among pig breeds [14]. Another research piece revealed a mutation in the *NR6A1* gene of European pigs, resulting in a change in the binding capacity of the NR6A1 protein and inhibitory proteins that interact with the NR6A1 protein [9]. Furthermore, polymorphisms in the *NR6A1* gene in Duroc pigs may affect body length [15]. It was confirmed that *NR6A1* is prominently associated with the number of vertebrae in Tongcheng (TC) pigs [16]. Similar results were found in Licha Black and Laiwu pigs [17]. More recently, a polymorphism in exon 8 of the *NR6A1* gene indicated the possible influence of this gene on the number of lumbar vertebrae in sheep [10].

In addition, the high homology of *NR6A1* among different species indicates its potential functional conservation [18]. Based on previous research [8,9,10,11,12,13,14,15,16,17,18], it is rational to hypothesize that the *NR6A1* gene may be associated with body size traits of other species. Presently, there is no research regarding the *NR6A1* gene in donkeys. Donkeys are significant domestic animals for meat production and other uses in China. In this research, we explored the polymorphism of the donkey *NR6A1* gene and its association with body size traits, especially body length and body height.

## 2. Materials and Methods

### 2.1. Ethics Statement

The protocols used in this study and for the animals were recognized by the Faculty of Animal Policy and Welfare Committee of Northwest A&F University (FAPWC-NWAFU, protocol number, NWAFAC1005).

### 2.2. Animals and Data Collection

The blood samples of 346 Dezhou female donkeys (3 years old) were collected from a donkey breeding farm (Dezhou, Shandong, China). Blood samples from 62 Guanzhong female donkeys (3 years old) from Shaanxi Agricultural and Animal Breeding Farm (Fufeng, Shaanxi, China) were collected for further investigation.

Nine body size traits (body height, chest measurement, chest width, rump length, thurl width, body length, rump height, chest depth, and cannon circumference) of 346 Dezhou donkeys, together with three body size traits (body weight, body height, and chest measurement) of 62 Guanzhong donkeys were recorded.

### 2.3. DNA Extraction and DNA Pool Construction

Genomic DNA was extracted from the blood samples following the phenolchloroform method [19]. DNA concentration and purity were estimated using NanoDrop ND 1000 Spectrophotometer (Thermo Fisher Scientific) and diluted to 50 ng/μL, then preserved at 4 °C.

### 2.4. PCR Amplification and Sequenci

The DNA pool containing 20 individual genomic DNA samples randomly chosen from each donkey breed was used as a template to carry out PCR amplification. Then, the PCR products of the DNA were sequenced in both the forward and reverse directions in order to identify the polymorphism. The primers were designed based on the DNA sequence of the donkey *NR6A1* gene in the NCBI database (Gene ID: 106830815). The primer pair was designed to amplify the target intron-1 (forward: 5’-ACCAAAAGCACAGTGCCTAGT-3’; reverse: 5’-TCCCAGAGTGCTAGGCTTGA. The final PCR amplification volume was kept at 12.5 μL, including 50 ng genomic DNA, 6.25 μL 2 × Taq PCR Master Mix (Kangwei century biotechnology co., LTD, Beijing, China), 4.75 μL ddH_2_O, and 0.5 μmol/L of each primer. The PCR protocol was as follows: 5 min at 95 °C, 10 cycles of touchdown at 65 °C, 20 cycles of 95 °C for 30 s, 60 °C for 30 s, and 72 °C for 30 s, with a final extension at 72 °C for 10 min. The PCR products were sent to a specialized sequencing company to complete the follow-up Sanger bi-directional sequencing (Shenggong, Shanghai, China). The sequencing results were analyzed using Bio XM (Ver. 2.6) software.

### 2.5. Genotyping

After sequencing the PCR products of the DNA pool, they were analyzed to determine the 13 bp deletion. This deletion was genotyped in 408 individuals via electrophoresis using 10% polyacrylamide gel (PAGE) at 220 volts for about 2 h.

### 2.6. Statistical Analysis

The results of the genotyping were statistically analyzed, and the genetic polymorphism index of the loci, including gene homozygosity (Ho), gene heterozygosity (He), effective allele numbers (Ne), and polymorphism information content (PIC), was estimated. The frequencies of the genotypes and alleles were calculated. A chi-square test was performed to verify whether allele frequency distribution conformed to the Hardy–Weinberg equilibrium. SPSS 23.0 software (Statistical Product and Service Solutions, Version23.0 Edition, IBM, Armonk, NY, USA) was used to analyze the association between genotypes and body size traits (body height, chest measurement, chest width, rump length, thurl width, body length, rump height, chest depth, and cannon circumference). The linear analysis model can be written as Y = μ + a + b + c, where Y is the observation of the growth trait, μ represents average deviation, a represents the fixed factor age, b represents the fixed factor genotype, and c is the random error.

## 3. Results

### 3.1. Sequence Variants Identified in the Donkey NR6A1 Gene

**Figure 1:**
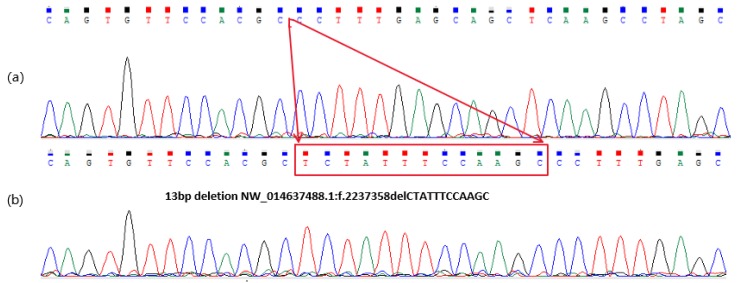
Chromatogram of the 13 bp deletion of the donkey *NR6A1* gene. (**a**) Homozygotic deletion/deletion type (DD) of the *NR6A1* locus; (**b**) homozygotic insertion/insertion type (II) of the *NR6A1* locus. The sequence with the red line boundary is the 13 bp deletion.

**Figure 2:**
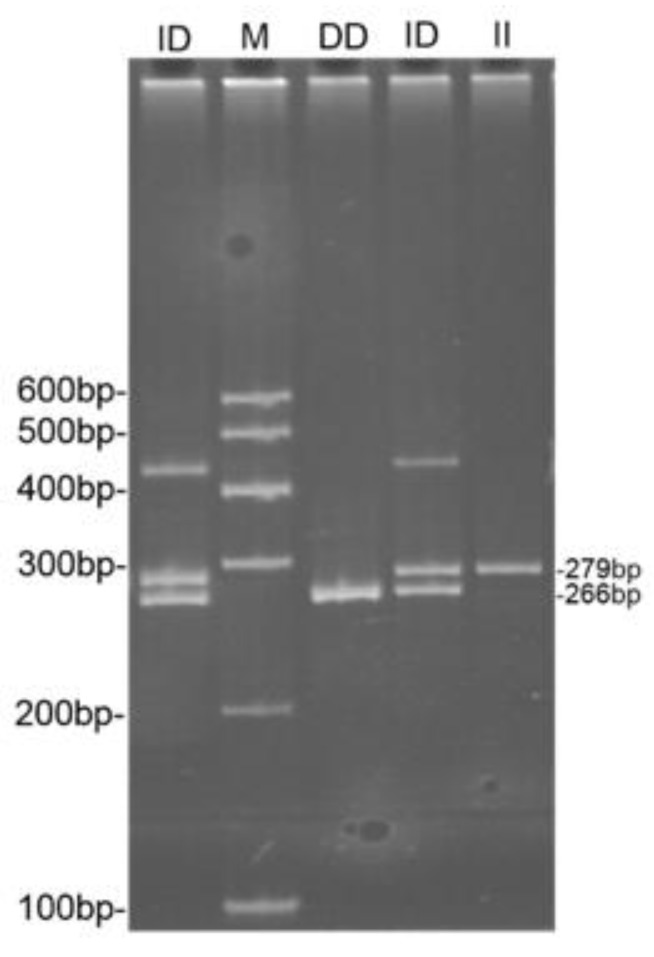
Polyacrylamide gel electrophoresis patterns of the deletion locus in the donkey *NR6A1* gene.

After PCR amplification and sequencing of the potential polymorphic locus, a 13 bp deletion was found, located in intron-1 of the *NR6A1* gene; the deletion sequence was TCTATTTCCAAGC. The sizes of the gene sequences generated by the sequencing were 279 bp and 266 bp. The sequencing results are shown in Figure 1 below. PCR amplification was performed on all samples at this site and polyacrylamide gel electrophoresis was used to genotype them. The results are shown in Figure 2. Genotype II was represented by a 279 bp fragment, genotype DD was represented by a 266 bp fragment, and genotype ID was represented by both 279 and 266 bp fragments. Three genotypes were randomly selected for sequencing verification, and these results were consistent with the results of the PAGE.

The PCR products showed three genotypes in the 13 bp deletion locus of the donkey *NR6A1* gene which was detected by 10% PAGE. *NR6A1*, the homozygote type (II genotype), showed a 279 bp fragment and the heterozygote type (ID genotype), showed two fragments (279 bp and 266 bp). The band around 450 bp was a heteroduplex, which probably formed due to the reannealing of the complementary strands as the DNA concentration changed, therefore outcompeting the hybridization of the oligonucleotides with their template strands [20].

### 3.2. Polymorphisms and Genetic Diversity

The statistical results of the genotypic and allelic frequencies in the 13 bp deletion loci of donkey *NR6A1* gene are shown in Table 1, in which the homozygous II genotype of the two breeds of donkey was the most common. The three genotypes, namely II, ID, and DD, were detected in both Dezhou donkeys and Guanzhong donkeys.

As shown in Table 1, II was the dominant genotype and I was the dominant allele. For the 13 bp deletion locus, the genotypes II, DD, and ID were detected in Dezhou donkeys. The frequencies of the II, DD, and ID genotypes were 0.84, 0.01, and 0.15, whereas the frequencies of the I and D alleles were 0.92 and 0.08, respectively. The frequency of the II, DD, and ID genotypes detected in Guanzhong donkeys were 0.82, 0.02, and 0.16, whereas the I and D allele frequencies were recorded as 0.90 and 0.10 respectively. The Hardy–Weinberg test showed that the genotype distribution observed corresponded with what was expected (*p* > 0.05), indicating that the colony was large and interbred freely.

Genetic parameter estimation showed that the PIC values of Dezhou donkeys and Guanzhong donkeys were 0.14 and 0.16, representing low polymorphisms (PIC < 0.25). The chi-square test showed that there were no significant differences in genotype frequencies and allele frequencies between the two breeds (Table 2).

### 3.3. Association Analysis of Polymorphisms with Growth Traits of the Donkey

For the 13 bp deletion locus, the association analysis between the polymorphic genotypes and body length, body height, chest circumference, cannon circumference, chest depth, chest width, rump height, rump width, and rump length in Dezhou donkeys showed that there were significant differences between the means of the II and ID genotypes for the body length (*p* = 0.025), body height (*p* = 0.019), chest circumference (*p* = 0.048), and chest depth (*p* = 0.002) in these donkeys (Table 3).

Furthermore, in Guanzhong donkeys, the body heights of genotype II individuals were significantly higher than genotype ID individuals (*p* = 0.048). Other traits were not significantly different (Table 3).

## 4. Discussion

Presently, the rapid information elevation in animal breeding and genetics has resulted from enhanced and increasingly accurate molecular tools. Using molecular markers to select genotypes of target traits can effectively improve economic benefits. In this study, we identified a 13 bp deletion in the *NR6A1* gene through DNA pool sequencing of two Chinese donkey breeds.

*NR6A1* expression is crucial for normal embryo formation at the embryonic development stage. Some studies showed that NR6A1 specifically recruited methylated CpG binding domaind and methyltransferase to the promoter of POU5F1, a marker molecule of undifferentiated embryonic stem cells (ESC), inhibiting the expression of *POU5F1* and playing an important role in the self-renewal and development of ESC [21,22]. In neural stem cells, *NR6A1* also directly targeted and inhibited NR6A1 expression, promoting the differentiation of neural stem cells and regulating the development of the nervous system [23].

The 13 bp deletion is located on intron-1 of the *NR6A1* gene. Although non-coding protein sequences, introns can be involved in regulating gene expression. For example, miRNA encoded by some introns affects the expression of a gene [24]. Certain introns contain transcriptional regulatory elements such as TATA boxed and CAAT boxed, which can regulate the activity of promoters and enhancers [25]. The variation in these intronic sequences might produce new splicing sites [26], leading to altered transcription products and ultimately affecting gene function. Subsequent association analysis showed that the 13 bp deletion was indeed associated with four important indicators of body size traits, including body height, body length, chest depth, and chest circumference. Many studies focused on these body size traits. For example, polymorphic loci significantly related to body length and height were found in the *UCP3* gene in Simmental Hybrid Cattle. Meanwhile [4], polymorphic loci that are significantly associated with body length and body size were also identified in the *ZBTB38* gene and the *NPY* gene in Nanyang, Qinchuan, and other Chinese cattle breeds [6,7]. In the present study, these four traits (body height, body length, chest depth and chest circumference) were significantly different between individuals with various genotypes in Dezhou donkey individuals. However, another important body size trait, body length, did not differ significantly between individuals with different genotypes in Guanzhong donkeys, perhaps due to the relatively lower sample size. In this study, an *NR6A1* gene polymorphism was investigated in donkeys for the first time, and it was found that the *NR6A1* gene polymorphism was significantly associated with donkey body size. Therefore, this could be used as a molecular marker to screen out individuals with better growth traits at an early stage to improve economic benefits and speed up the breeding process of donkeys.

## 5. Conclusions

The donkey raising industry is characteristic of animal farming in China, but there is a lack of specialized breeds, which restricts the development of the industry. In this study, we focused on the important economic character of donkey body size traits. Based on the candidate gene method, a 13 bp deletion was found in an intron of the donkey *NR6A1* gene, which was significantly associated with body size traits, especially body length and body height, of Guanzhong and Dezhou donkeys. This gene could be used as a potential molecular marker for growth traits and a candidate molecular marker for body trait selection, which is of great significance to the development of the donkey industry.

## Figures and Tables

**Table 1:** Genotypic and allelic frequencies, gene heterozygosity (He), effective allele numbers (Ne), polymorphism information content (PIC) and Hardy–Weinberg equilibrium in the 13 bp deletion locus of the donkey *NR6A1* gene.

Locus	Breeds	N	Genotypic Frequencies			Allelic Frequencies		HWE	Population Parameters			
			II	ID	DD	I	D	*p*	Ho	He	Ne	PIC
*NR6A1* *(NW-014637488)*	DZ	346	0.84 (*n* = 291)	0.15 (*n* = 53)	0.01 (*n* = 2)	0.92	0.08	0.81	0.849	0.151	1.178	0.14
	GZ	62	0.82 (*n* = 51)	0.16 (*n* = 10)	0.02 (*n* = 1)	0.90	0.10	0.54	0.825	0.175	1.212	0.16

InDel—insertions/deletions; N—number; HWE—Hardy–Weinberg equilibrium; Ho—homozygosity; He—heterozygosity; Ne—effective allele numbers; PIC—polymorphism information content; DZ—Dezhou donkey; GZ—Guanzhong donkey; II—insertion/insertion; ID—insertion/deletion; DD—deletion/deletion.

**Table 2:** χ^2^ test of different breeds on the 13 bp deletion locus of the donkey *NR6A1* gene.

Locus	Types	Breeds	DZ	GZ
*NR6A1 (NW-014637488)*	Genotypic frequencies	DZ	–	χ^2^ = 0.81
		GZ	(*p* > 0.05)	–
	Allelic frequencies	DZ	–	χ^2^ = 0.06
		GZ	(*p* > 0.05)	–

Indel—insertions/deletions; DZ—Dezhou donkey; GZ—Guanzhong donkey.

**Table 3:** Relationship between the 13 bp deletion locus of the donkey *NR6A1* gene and growth traits with the same genotype (II, ID, and DD genotypes, respectively).

Locus	Breeds	Growth Traits	Observed Genotypes (LSM ^a^ ± SE)			*p*
			II	ID	DD	
*NR6A1* *(NW-014637488)*	DZ	Body height	136.56 ± 6.94 ^a^ (*n* = 291)	134.98 ± 5.11 ^b^ (*n* = 53)	133.00 ± 7.07 (*n* = 2)	0.019
		Body length	135.78 ± 8.17 ^a^ (*n* = 291)	135.32 ± 6.38 ^b^ (*n* = 53)	133.00 ± 5.65 (*n* = 2)	0.025
		Chest circumference	148.66 ± 8.89 ^a^ (*n* = 291)	148.11 ± 6.90 ^b^ (*n* = 53)	157.75 ± 10.96 (*n* = 2)	0.048
		Chest depth	55.26 ± 3.86 ^a^ (*n* = 291)	54.83 ± 2.61 ^b^ (*n* = 53)	56.00 ± 4.24 (*n* = 2)	0.002
	GZ	Body height	135.68 ± 6.77 ^a^ (*n* = 51)	133.10 ± 4.53 ^b^ (*n* = 10)	0	0.048
		Body length	128.77 ± 7.07 ^a^ (*n* = 51)	125.70 ± 5.27 ^a^ (*n* = 10)	0	0.467

NR6A1—Nuclear Receptor Subfamily 6 group member1; LSM—least squares mean; SE—standard error; DZ—Dezhou donkey; GZ—Guanzhong donkey. ^a,b^ Means with different letters differed significantly (*p* < 0.05). Individuals of the DD genotype were excluded from this analysis due to the limited number (*n* = 2).
